# Effects of xenon anesthesia on postoperative neurocognitive disorders: a systematic review and meta-analysis

**DOI:** 10.1186/s12871-023-02316-5

**Published:** 2023-11-09

**Authors:** Yu-Shen Yang, Shan-Hu Wu, Wei-Can Chen, Meng-Qin Pei, Yi-Bin Liu, Chu-Yun Liu, Shu Lin, He-Fan He

**Affiliations:** 1 Department of Anaesthesiology, the Second Affiliated Hospital of Fujian Medical University, Quanzhou, China; 2 Centre of Neurological and Metabolic Research, the Second Affiliated Hospital of Fujian Medical University, Quanzhou, China; 3 Neuroendocrinology Group, Garvan Institute of Medical Research, Darlinghurst, Australia

**Keywords:** Meta-analysis, Postoperative neurocognitive disorders, Randomized clinical trial, Systematic review, Xenon anesthesia

## Abstract

The latest clinical trials have reported conflicting outcomes regarding the effectiveness of xenon anesthesia in preventing postoperative neurocognitive dysfunction; thus, this study assessed the existing evidence. We searched the PubMed, Embase, Cochrane Library, and Web of Science databases from inception to April 9, 2023, for randomized controlled trials of xenon anesthesia in postoperative patients. We included English-language randomized controlled studies of adult patients undergoing surgery with xenon anesthesia that compared its effects to those of other anesthetics. Duplicate studies, pediatric studies, and ongoing clinical trials were excluded. Nine studies with 754 participants were identified. A forest plot revealed that the incidence of postoperative neurocognitive dysfunction did not differ between the xenon anesthesia and control groups (*P* = 0.43). Additionally, xenon anesthesia significantly shortened the emergence time for time to opening eyes (*P* < 0.001), time to extubation (*P* < 0.001), time to react on demand (*P* = 0.01), and time to time and spatial orientation (*P* = 0.04). However, the Aldrete score significantly increased with xenon anesthesia (*P* = 0.005). Postoperative complications did not differ between the anesthesia groups. Egger’s test for bias showed no small-study effect, and a trim-and-fill analysis showed no apparent publication bias. In conclusion, xenon anesthesia probably did not affect the occurrence of postoperative neurocognitive dysfunction. However, xenon anesthesia may effectively shorten the emergence time of certain parameters without adverse effects.

**Supplementary Information:**

The online version contains supplementary material available at 10.1186/s12871-023-02316-5.

## Introduction

Postoperative neurocognitive disorders (PNDs) are common postoperative complications in older patients, with an incidence of 41–75% at seven days postoperatively [1]. Based on the onset time, PNDs can be divided into postoperative acute delirium, generally occurring within hours to days after anesthesia and surgery, and postoperative cognitive dysfunction, which generally occurs within weeks to months after surgery) [2]. The clinical manifestations of PNDs include language, learning, thinking, memory, emotional, and spirit disorders, as well as reduced cognitive function, which can lead to prolonged hospitalization, high costs, poor quality of life, increased postoperative mortality, and a heavy social burden [2, 3]. Therefore, it is crucial to develop safe and effective strategies to reduce the occurrence of PND.

Xenon is a monoatomic inhalational agent that has been shown to protect neurons from damage in animal models [4]. In recent years, xenon has been considered a better inhalational anesthetic agent for older surgical patients because of its hemodynamic stability and cytoprotective properties [5]. Xenon has also been reported to be crucial in reducing the incidence of PND in surgical patients [6, 7]. However, the latest clinical trials have reported conflicting outcomes. Al Tmimi et al. reported that xenon anesthesia did not significantly reduce the incidence of PND; thus, they did not recommend xenon for PND prevention [8]. Similarly, Coburn et al. performed a multicenter, randomized clinical trial including 256 patients undergoing hip fracture surgery, demonstrating that xenon anesthesia did not decrease the occurrence of PND following surgery [9].

Therefore, this systematic review and meta-analysis analyzed randomized clinical trials to investigate the effectiveness of xenon in preventing PND in anesthetized and surgical patients.

## Methods

The current systematic review and meta-analysis was conducted following the Guidelines of Preferred Reporting Items for Systematic Reviews and Meta-Analyses. A specialist team that included an anesthetist, neurologist, and methodologist formulated clinical questions and provided input on the study protocol. The PROSPERO registration number is CRD42022329958.

### Systematic literature search

Qualified randomized clinical trials were extracted from the following databases: PubMed, Embase, Cochrane Library, and Web of Science databases (all dated until April 9, 2023) by two independent authors. Detailed search strategies and results for databases used by this study can be found in Additional file 1. Furthermore, relevant recent reviews and reference lists of all randomized clinical trials were retrieved. Additionally, we reviewed conference abstracts of major societies over the past three years.

### Inclusion and exclusion criteria

The inclusion criteria were: (1) Participants: patients undergoing surgery; (2) Intervention: xenon anesthesia; (3) Comparison: other inhalation or intravenous anesthetics; (4) Outcomes: studies reporting the effects of xenon anesthesia; (5) Study design: studies designed as clinical randomized clinical trials; and (6) Language: limited to randomized clinical trials conducted in humans and publications in English, as the quality of studies conducted in other languages could not be adequately assessed.

Studies were excluded based on the following criteria: (1) ongoing clinical trials; (2) pediatric patients; (3) duplicate publications and reports from the same trial; (4) case report; (5) without available outcomes.

### Data extraction

EndNote X9 (Clarivate, London, UK) was used to exclude duplicates. Two researchers (YSY and SHW) independently checked the article titles, abstracts, or full texts to determine their eligibility. A third researcher (HFH) resolved any differences between the two authors. Two researchers (YSY and SHW) independently extracted the following data from eligible studies: first author name, year of publication, age, sample size, American Society of Anesthesiologists physical status, type of surgery, xenon dose, comparison, and PND assessment method.

### Quality and risk assessment

Two researchers assessed the methodological quality and risk of bias of included trials based on the revised Cochrane risk of bias tool for randomized trials, which covered the following domains: random sequence generation, allocation concealment, blinding of participants and personnel, blinding of outcome assessment, incomplete outcome data, selective reporting, and other bias [10, 11]. The level of certainty was determined using the Grading of Recommendations, Assessment, Development, and Evaluation (GRADE) system, with results classified as high, moderate, low, or very low.

### Primary and secondary outcomes

The primary outcome of this meta-analysis was the incidence of PND. Secondary outcomes included the results of the postoperative cognitive evaluation, time to opening eyes, extubation time, Aldrete score, time to react on demand, time to time and spatial orientation, and postoperative adverse events (sepsis, respiratory infection or inflammation, acute kidney injury, myocardial dysfunction/infarction, hypotension, postoperative nausea and vomiting [PONV], and mortality). Given the heterogeneity of the PND assessment methods, we planned a priori to accept assessment results reported by similar methods (e.g., Mini-Mental State Examination [MMSE], alertness, divided attention, and working memory). All outcome definitions per study are detailed in Additional file 2.

### Statistical analyses

All meta-analyses were performed using Review Manager (version 5.4; The Cochrane Collaboration, London, UK) and STATA V.12.0 (StataCorp, College Station, TX, USA). Pooled risk ratios (RRs) and 95% confidence intervals (CIs) were calculated for dichotomous variables. *P* < 0.05 was used to determine a statistically significant result. Mean differences and 95% CIs were calculated for continuous variables in the same units. For continuous variables described as means (95% CIs), we shifted to means and standard deviations per the Cochrane Handbook for Systematic Reviews of Interventions (version 6.3). The heterogeneity of trials was evaluated using the I^2^ statistic. Furthermore, sensitivity analysis using a subset design was conducted to evaluate the reliability and robustness of the effect estimate. A subgroup analysis was performed based on the different surgery methods (cardiac surgery vs. orthopedic surgery). However, high clinical heterogeneity usually comes from various methodological and clinical factors. Thus, a random-effects model was used despite the low I^2^ value.

The small-study effect and publication bias were assessed using an Egger’s test and trim-and-fill analysis. Viewer software (version 0.9.5.10 Beta) was used to perform trial sequential analysis for the primary outcome to demonstrate whether firm evidence was reached. Finally, in order to correct for the incremental risk of type I errors, trial sequential analysis (TSA) was employed to identify whether the findings of the cumulative meta-analysis were reliable and conclusive. TSA combines the required information size (RIS) with the trial sequential monitoring boundary to adjust CI and reduce type I errors [12]. When the z-curve dose not traverse the trial sequential monitoring boundary or enters the futility area, the evidence is considered inadequate to derive conclusions, and thus further studies are required. If the boundary is crossed by the z-curve and the RIS has been reached, dependable and conclusive evidence has been obtained. Trial sequential analysis version 0.9 beta145 (http://www.ctu.dk/tsa) was used for all these analyses.

## Results

### Search results

In total, 1855 relevant studies were initially obtained from the databases. Based on the inclusion and exclusion criteria, 326 duplicated publications and 1501 studies were removed after reading the abstracts and titles. This left 28 preliminarily qualified trials after evaluating their full text; however, nineteen were excluded based on the following reasons: pediatric patients (*n* = 4) [13–16], case report (*n* = 1) [17] and lack of available outcome (*n* = 14) [18–31]. Finally, nine studies [6–9, 32–36] met the inclusion criteria and were included in the meta-analysis (see Additional file 3).

### Study characteristics and risk of bias

Table 1 presents the characteristics of the qualified studies. The current meta-analysis included nine randomized clinical trials with a total of 754 patients; 374 patients received xenon, and 380 received a control. The publication years varied from 2006 to 2020, the study sample sizes ranged from 30–256, patient age ranged from 18–98.5 years old, and the American Society of Anesthesiologists physical status was I–VI. The types of surgeries included orthopedic surgery [9, 32], cardiac surgery [7, 8, 36], and other elective surgery [6, 33–35]. Sevoflurane-based general anesthesia was used in six studies [6–9, 35, 36], whereas other anesthetics (propofol [32], desflurane [33], and isoflurane [34]) were used in one study. PND was assessed using the Confusion Assessment Method in four randomized clinical trials [7–9, 36], whereas other studies used the neuropsychological test battery for the International Study of Postoperative Cognitive Dysfunction [32], Test for Attentional Performance [33, 35], Short Orientation Memory Concentration Test [6], and Syndrome Short Test [34]. Additional file 4 presents the risk of bias results.

**Table 1:** The details of the included studies

Author	Age (years)	ASA scale	Type of surgery	Intervention	Sample size (n)	Control	Sample size (n)	PND assessment
Rasmussen 2006 [32]	≥ 60	-	Knee replacement	60–70% xenon	21	Intravenous propofol (3–5 mg kg^−1^ h^−1^)	18	ISPOCD neuropsychological test battery
Coburn 2007 [33]	65–75	I–III	Elective surgery^a^	60% xenon	18	5.2–5.5% desflurane anesthesia	20	Test for Attentional Performance
Bronco 2010 [6]	42–74	I-II	Elective surgery^b^	60% xenon	29	1.4% Sevoflurane	30	Short Orientation Memory Concentration Test
Stuttmann 2010 [34]	≥ 18	I-II	Elective surgery^c^	63% xenon	31	0.6% isoflurane	30	Syndrome short test
Cremer 2011 [35]	65–75	I-III	Elective surgery^d^	60% xenon	19	1.1–1.4% Sevoflurane	20	Test of Attentional Performance
Stoppe 2013 [36]	48–81	II–IV	Elective CABG surgery	45–50% xenon	15	1–1.4% Sevoflurane	15	Confusion Assessment Method
Al tmimi 2015 [7, 23]	47–86	III-IV	Elective OPCAB surgery	50–60% xenon	21	1.1–1.4% Sevoflurane	21	Confusion Assessment Method
Coburn 2018 [9]	≥ 75	I-III	Hip fracture surgery	60% xenon	124	1.1–1.4% Sevoflurane	132	Confusion Assessment Method
Al tmimi 2020 [8]	≥ 65	III-IV	On-pump cardiac surgery	40–60% xenon	96	1.1–1.4% Sevoflurane	94	Confusion Assessment Method

*Abbreviations*: *ASA* American Society of Anesthesiologists physical status, *CABG* coronary artery bypass grafting, *PND* Postoperative neurocognitive dysfunction, *OPCAB* Off-pump coronary artery bypass. The types of elective surgery were as follows

^a^surgery in trauma, ear, nose, and throat, gynecology and urology

^b^visceral surgical strumectomy, augmentation or reduction mammaplasty, liposuction in obese patients and knee arthroscopy

^c^general surgery, ear nose and throat surgery, gynecological surgery, orthopedic surgery and urological surgery

^d^urology, gynecology, neurosurgery, trauma, ENT, orthopedics and abdominal surgery

### Outcomes

#### Incidence of PND

**Fig. 1:**
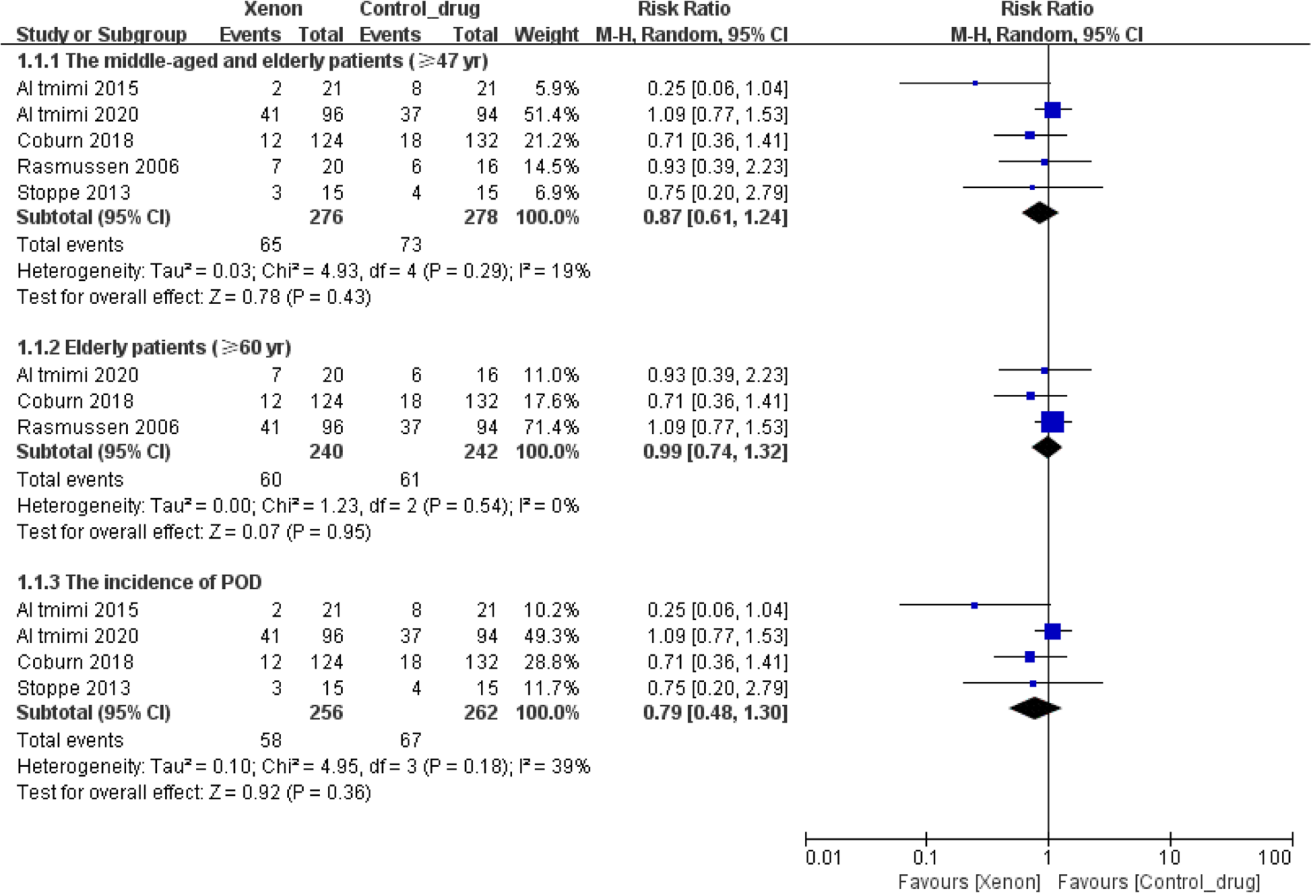
Forest plot of the pooled analysis of the incidence of postoperative neurocognitive disorders

Five trials reported the occurrence of PND comprising 554 patients (≥ 47 years old). The comprehensive forest plot results showed that xenon did not affect the incidence of PND (RR = 0.87, 95% CI 0.61 to 1.24; *P* = 0.43, I^2^ = 19%, Fig. 1). A similar phenomenon was observed in older patients (≥ 60 years old) (RR = 0.99, 95% CI 0.74 to 1.32; *P* = 0.95, I^2^ = 0%, Fig. 1) and POD patients (RR = 0.79, 95% CI 0.48 to 1.30; *P* = 0.36, I^2^ = 39%, Fig. 1). Moreover, the subgroup analysis results were consistent with the overall results (see Additional file 5). A sensitivity analysis on the incidence of PND revealed that the effect estimate remained unchanged (see Additional file 6).

#### PND assessments

**Fig. 2:**
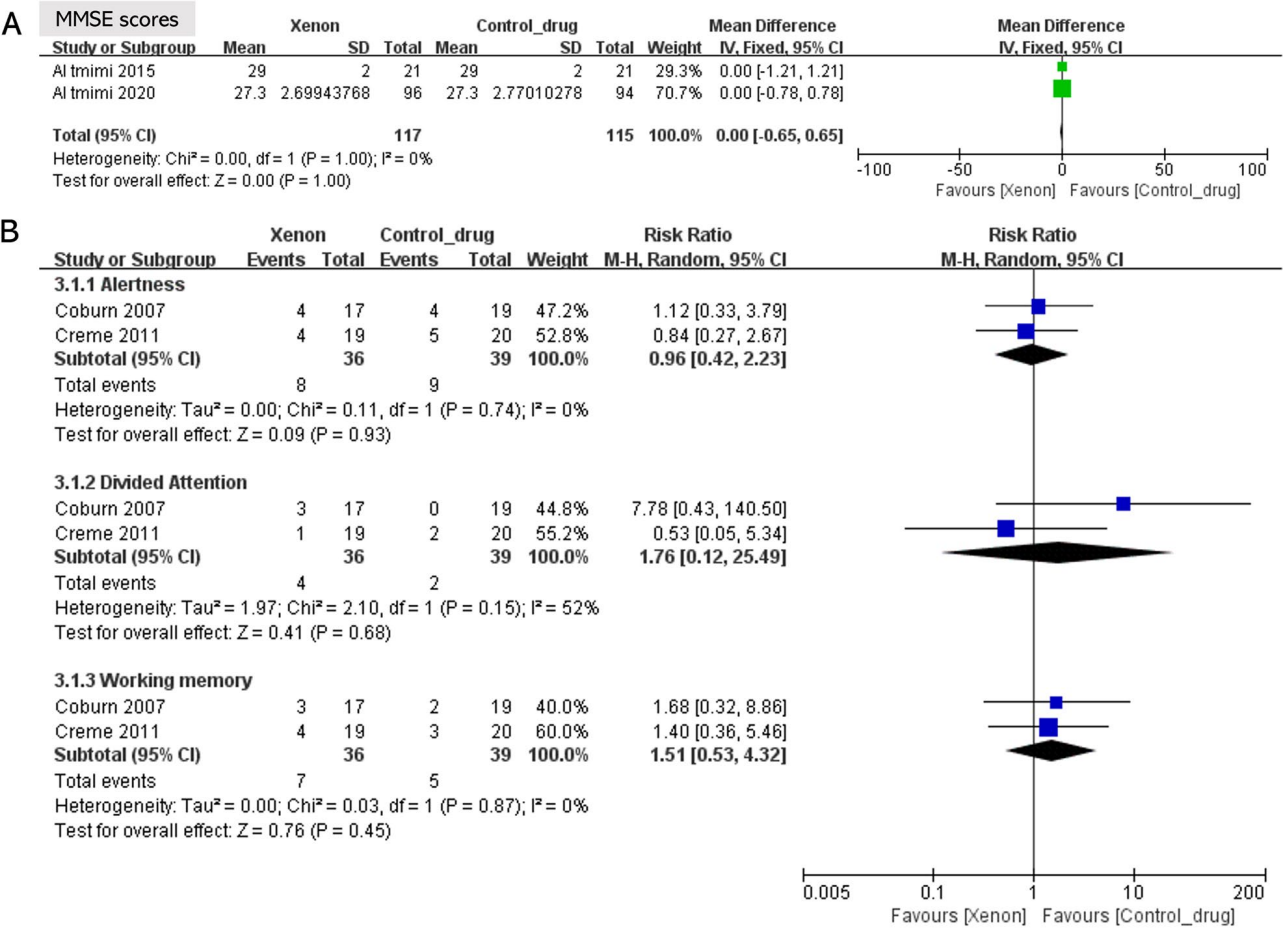
Forest plot of the pooled analysis of the postoperative cognitive evaluation results

MMSE scores were recorded in two trials after surgery. The MMSE scores did not differ between the two groups in these studies (forest plot; mean difference = 0.00, 95% CI –0.65 to 0.65; *P* = 1.0, I^2^ = 0%; Fig. 2A). Two trials recorded the results of the Test of Attentional Performance after surgery (alertness, RR = 0.96, 95% CI 0.42 to 2.23, *P* = 0.93, I^2^ = 0%; divided attention, RR = 1.76, 95% CI 0.12 to 25.49, *P* = 0.68, I^2^ = 52%; working memory, RR = 1.51, 95% CI 0.53 to 4.32, *P* = 0.45, I^2^ = 0%; Fig. 2).

#### Emergence variables

**Fig. 3:**
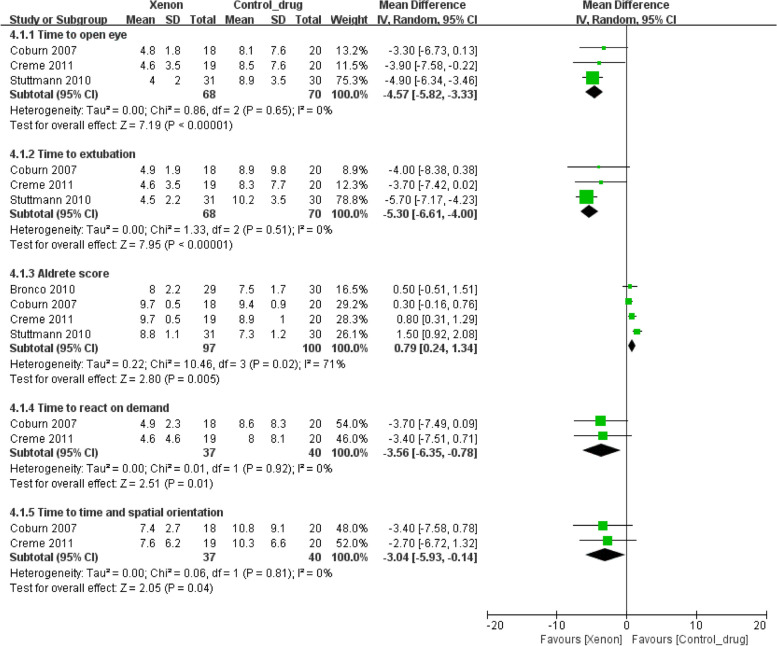
Forest plot of the pooled analysis of the emergence parameters from anesthesia

Three studies presented results of the time to opening eyes and time to extubation, four studies reported the Aldrete score, and two studies reported the time to react on demand and time to time and spatial orientation. Forest plots showed that xenon significantly decreased the time to opening eyes, time to extubation, time to react on demand, and time to time and spatial orientation. However, xenon increased the Aldrete score (time to opening eyes, mean difference = –4.57, 95% CI –5.82 to –3.33, *P* < 0.001, I^2^ = 0%; time to extubation, mean difference = –5.30, 95% CI –6.61 to –4.00, *P* < 0.001, I^2^ = 0%; Aldrete score, mean difference = 0.79, 95% CI 0.24 to 1.34, *P* = 0.005, I^2^ = 71%; time to react on demand, mean difference = –3.56, 95% CI –6.35 to –0.78, *P* = 0.01, I^2^ = 0%; time to time and spatial orientation, mean difference = –3.04, 95% CI –5.93 to –0.14, *P* = 0.04, I^2^ = 0%; Fig. 3).

#### Adverse effects

Three trials reported sepsis and acute kidney injury, and three others reported PONV; the incidence rates did not differ between the two groups (forest plots; sepsis, RR = 0.96, 95% CI 0.27 to 3.40, *P* = 0.95, I^2^ = 37%; acute kidney injury, RR = 1.07, 95% CI 0.53 to 2.17, *P* = 0.85, I^2^ = 5%; PONV, RR = 1.20, 95% CI 0.67 to 2.15, *P* = 0.54, I^2^ = 0%). The incidence of other complications did not differ between the two groups (respiratory infection/inflammation, RR = 0.62, 95% CI 0.30 to 1.32, *P* = 0.22, I^2^ = 0%; myocardial dysfunction/infarction, RR = 0.45, 95% CI 0.13 to 1.59, *P* = 0.21, I^2^ = 0%; hypotension, RR = 0.89, 95% CI 0.65 to 1.22, *P* = 0.46, I^2^ = 0%; mortality, RR = 0.81, 95% CI 0.08 to 8.64, *P* = 0.86, I^2^ = 52%; Additional file 6). However, a meta-analysis of desaturation has not been performed because of an insufficient number of trials.

### Small-study effect and publication bias

**Fig. 4:**
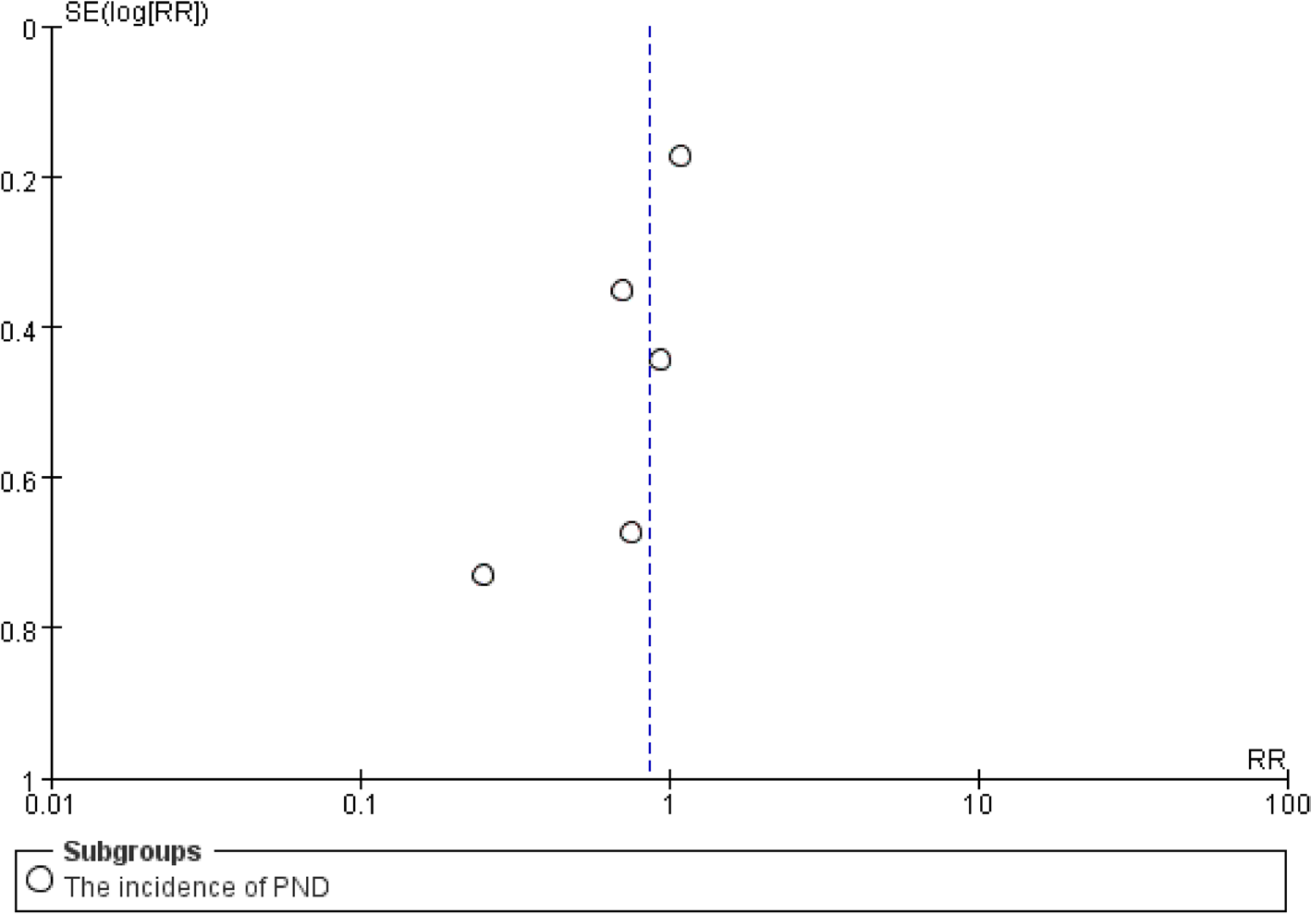
Funnel plot of risk ratio (x axis) by standard error (y axis)

The bias and 95% CI of Egger’s test contained 0 (bias = –1.65, 95% CI –4.73 to 1.43, *P* = 0.19, *P* = 0.33), which showed no small-study effect. The trim-and-fill analysis results also did not show obvious publication bias (see Additional file 7). In addition, funnel plot looks reasonably symmetrical, which also supports the results of egger’s test and trim-and-fill analysis (Fig. 4).

### Trial sequential analysis and GRADE assessment

**Fig. 5:**
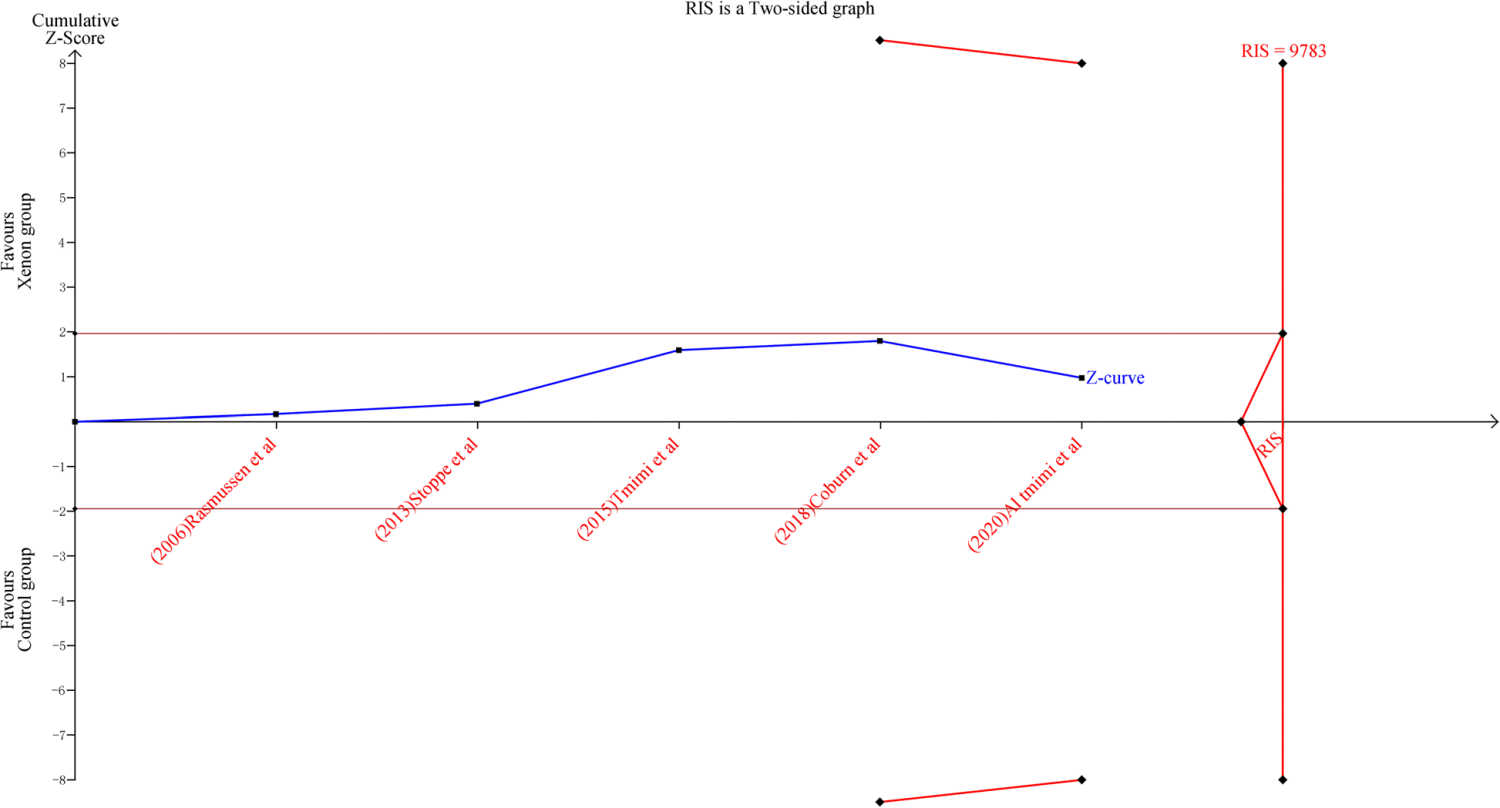
The result of trial sequential analysis. RIS, required information size

The trial sequential analysis results indicate that the required information size is 9783; therefore, firm evidence was not obtained regarding xenon’s neutral effect on perioperative cognitive function (Fig. 5). Thus, more studies are needed to confirm the neuroprotective effect of xenon anesthesia. In addition, based on the GRADE system, the quality of evidence for the primary and secondary outcomes ranged from low to high (Table 2).

**Table 2:** The overall results of GRADE evaluation

Outcome	MD/RR [95%CI]	*I*^2^	Quality of evidence	Reasons
The incidence of PND	0.87 [0.61, 1.24]	19%	⨁⨁⨁⨁ HIGH	None
MMSE	0.00 [-0.66, 0.66]	0%	⨁⨁⨁⨁ HIGH	None
Alertness	0.96 [0.42, 2.23]	0%	⨁⨁⨁⨁ HIGH	None
Divided Attention	1.76 [0.12, 25.49]	52%	⨁⨁◯◯ LOW	Inconsistency was “serious” ^a^, imprecision was “serious”^b^
Working Memory	1.51 [0.53, 4.32]	0%	⨁⨁⨁◯ MODERATE	Imprecision was “serious”
To open eyes	-4.57 [-5.82, -3.33]	0%	⨁⨁⨁◯ MODERATE	Indirectness was “serious” ^c^
To extubation	-5.30 [-6.61, -4.00]	0%	⨁⨁⨁◯ MODERATE	Indirectness was “serious”
Aldrete score	0.79 [0.24, 1.34]	71%	⨁⨁⨁◯ MODERATE	Inconsistency was “serious”
to react on demand	-3.56 [-6.35, -0.78]	0%	⨁⨁◯◯ LOW	Indirectness was “serious”, imprecision was “serious”
To time and spatial orientation	-3.04 [-5.93, -0.14]	0%	⨁⨁◯◯ LOW	Indirectness was “serious”, imprecision was “serious”
Sepsis	0.96 [0.27, 3.40]	37%	⨁⨁⨁◯ MODERATE	Inconsistency was “serious”
Respiratory infection/ inflammation	0.62 [0.30, 1.32]	0%	⨁⨁⨁⨁ HIGH	None
Acute kidney injury	1.07 [0.53, 2.17]	5%	⨁⨁⨁⨁ HIGH	None
Myocardial dysfunction/infarction	0.45 [0.13, 1.59]	0%	⨁⨁⨁⨁ HIGH	None
Hypotension	0.89 [0.65, 1.22]	0%	⨁⨁⨁⨁ HIGH	None
PONV	1.20 [0.67, 2.15]	0%	⨁⨁⨁⨁ HIGH	None
Mortality	0.81 [0.08, 8.64]	52%	⨁⨁◯◯ LOW	Inconsistency was “serious”, imprecision was “serious”

*CI* Confidence intervals, *PND* Postoperative neurocognitive disorders, *PONV* postoperative nausea and vomiting, *MD* Mean difference, *MMSE* Mini-mental state examination scores *RR* Risk ratio

^a^I^2^ > 30%, which indicated “inconsistency”, was graded as “serious”

^b^for outcomes have a wide confidence interval (gap > 3), we downgraded the level of certainty to “serious” for “imprecision”

^c^the results were reported as mean (95%CI), which indicated “indirectness” was classified as “serious”

## Discussion

This systematic review and meta-analysis demonstrated that xenon anesthesia did not affect the incidence of PND and postoperative cognitive scores in surgical patients. However, xenon anesthesia significantly shortened the emergence time for eye-opening time, extubation time, on-demand reaction time, and time and spatial orientation time as well as increased the Aldrete score. Finally, the incidence of postoperative complications did not differ between the two anesthesia groups, and the degree of certainty of GRADE varied from low to high.

PND has been described as a postoperative cognitive and psychiatric disorder that may manifest as anxiety, psychosis, memory impairment, and personality changes [2]. Although the pathogenesis of PND remains unclear, education level, age, anesthesia duration, severity of surgery, previous cognitive impairment, occurrence of complications, and increased blood pressure fluctuation during the operation are generally considered PND risk factors [37–40]. Some strategies have been designed to prevent the occurrence of PND. Yang et al. showed that intraoperative anesthesia depth monitoring improved PND and brain functional connectivity by inhibiting systemic inflammation [41]. Likewise, propofol in cardiac surgery effectively improves PND without increasing side effects [42]. In addition, dexmedetomidine reduces the incidence of PND after major surgery without increasing adverse effects [43]. Other approaches, such as intravenous anesthesia, multimodal analgesia, and intraoperative body temperature and blood pressure management, may also be helpful; however, clinical studies have presented conflicting results. For instance, some studies have demonstrated that intraoperative blood pressure management [44], propofol [45], dexmedetomidine [46], and the anesthesia type [47] may not be as effective as expected in reducing the incidence of PNDs. Therefore, the development and administration of drugs with minimal impact on cognitive function is important for this surgical population.

Xenon is an inert gas that does not undergo metabolism or biotransformation in the body. Thus, xenon protects neurons from ischemic injury by reducing neuronal excitability through activating plasma adenosine triphosphate-sensitive potassium channels. In addition, xenon is less neurotoxic in animal models [48, 49]. Based on these properties, xenon is suitable for patients at higher risk for PND [50].

A recent meta-analysis by Siu-Chun Law et al. reported that xenon might be associated with better neurological outcomes compared with the standard care therapy in specific clinical situations [51]. However, xenon’s efficacy for preventing PND has not been investigated in detail. We found that xenon does not influence the incidence of PND, which was confirmed in subgroup analyses for different surgery methods. Moreover, xenon significantly reduced the emergence times, such as the time to opening eyes, to extubation, to react on demand, and to time and spatial orientation. Additionally, we found significantly higher Aldrete score values in the xenon group than in the control group. A similar phenomenon was reported by Hou et al. in their systematic review and meta-analysis [52]. The lower blood-gas partition coefficient of xenon (0.115) compared to other inhaled anesthetics (sevoflurane, 0.69; isoflurane, 1.41) may explain this [53]. Thus, xenon contributes to the fast emergence from anesthesia. However, faster awakening is not necessarily related to a faster discharge from a post-anesthesia care unit (PACU), though it may help in the evaluation and care in the PACU. Discharge time mainly depends on perioperative complication variables, including bleeding, infection, pain, and PONV. Hence, we further evaluated the perioperative complications in both groups.

As an antagonist at the 5-HT3 receptor, xenon might exert antiemetic properties [54]. Recently, an observational study demonstrated that the incidence of PONV after xenon anesthesia was obviously lower than that predicted by the Apfel score [55]. However, two randomized clinical studies observed a contradictory phenomenon, reporting that the incidence of PONV following xenon anesthesia is significantly higher than that after sevoflurane [56] and propofol [57]. For this reason, some scholars regard the higher incidence of PONV to be a major limitation of xenon [5]. Nevertheless, in the present meta-analysis, a remarkable difference was not observed in the occurrence of PONV between the anesthesia groups. Furthermore, when evaluating the incidence of other adverse effects, patients anesthetized by xenon did not have significantly higher rates of sepsis, respiratory infection or inflammation, acute kidney injury, myocardial dysfunction/infarction, hypotension, or mortality compared with other narcotics. These results indicate that xenon probably has similar safety to other narcotic drugs. However, due to insufficient data, these findings must be accepted critically because the incidence of some complications was completely different from previous reports. For example, Coburn et al. indicated that the use of xenon is associated with a higher incidence of PONV compared with propofol [57]. Thus, the safety of xenon needs to be evaluated in the future in large size, multiple centers, randomized trial.

This meta-analysis had several limitations. First, according to the results of the trial sequential analysis, the included sample size of this study was small, though we systemically searched the databases. Second, this study included different types of surgeries, but most were cardiac and orthopedic surgeries. Third, this study only analyzed xenon concentrations of 40–70%. Fourth, the medication and anesthetic choices were not standardized. Fifth, subgroup analyses for different age groups could not be performed due to insufficient data; however, when only older patients were selected for further analysis, the results were probably consistent with the overall results. Sixth, a significantly number of studies were not included due to the exclusion criteria bounded by the primary outcome variables/primary intention of this study. This resulted in an inadequate search and inaccurate conclusion, which probably causes a misunderstanding regarding the effect of xenon on secondary outcomes. Finally, the results from the current study are emerging data, and when future high-quality randomized clinical trials are reported in the field, reappraisal is required for these data.

In summary, although current evidence suggests that administering xenon anesthesia probably does not affect the occurrence of PND in surgical patients compared to controls, there is inconclusive or insufficient data to further prove or disprove it. Meanwhile, it can significantly shorten the emergence time without other adverse reactions, but the availability at hospitals and cost restricts the use of xenon as an anesthetic drug of choice. Thus, xenon anesthesia seemingly does not show enough advantages in clinical application. If these drawbacks are overcome, the feasibility of xenon anesthesia over conventional volatile anesthetics in surgery could be further explored.

### Supplementary Information

**Additional file 1.** Search strategies for databases including PubMed, Embase, Cochrane, and Web of Science.

**Additional file 2.** Author's definition of each outcome and the anaesthesia induction program.

**Additional file 3.** Flow diagram of the literature search.

**Additional file 4.** Risk of bias (ROB 1.0) evaluations for the included randomized-controlled trials. Risk of bias (ROB 2.0) assessment of included randomized controlled trials.

**Additional file 5.** Forest plot of the pooled analysis showing the subgroup analysis for the incidence of PND according to different surgery types (PND, postoperative cognitive dysfunction).

**Additional file 6.** The results of sensitivity analysis.

**Additional file 7.** Forest plot of the pooled analysis of postoperative complications.

**Additional file 8.** The result of trim and fill analysis for the incidence of postoperative neurocognitive disorders.

## Data Availability

The datasets used and/or analyzed during the current study are available from the corresponding author on reasonable request.

## Abbreviations

CI: Confidence intervals
GRADE: Grading of Recommendations, Assessment, Development, and Evaluation
MMSE: Mini-Mental State Examination
PACU: Post-anesthesia care unit
PNDs: Postoperative neurocognitive disorders
PONV: Postoperative nausea and vomiting
RRs: Risk ratios
