# Supplementary material for: Effects of xenon anesthesia on postoperative neurocognitive disorders: a systematic review and meta-analysis
Source: BMC Anesthesiol. 2023 Nov 9;23:366. doi: 10.1186/s12871-023-02316-5 (PMC10634138; doi:10.1186/s12871-023-02316-5)
Supplement: Supplementary file 1 — Additional file 1. Search strategies for databases including PubMed, Embase, Cochrane, and Web of Science. [file 12871_2023_2316_MOESM1_ESM.docx]

**Additional file 1:** Search strategies for databases including PubMed, Embase, Cochrane, and Web of Science

1. **PubMed**

| **No.** | **Query** | **Results** |
| --- | --- | --- |
| #8 | #1 OR #2 OR #3 OR #4 OR #5 OR #6 AND #7 | 441 |
| #7 | (xenon[MeSH Terms]) OR (xenon[Title/Abstract]) | 12,569 |
| #6 | (((((((((((((((((Mental Disorders[MeSH Terms]) OR (Mental Disorder[Title/Abstract])) OR (Psychiatric Illness[Title/Abstract])) OR (Psychiatric Illnesses[Title/Abstract])) OR (Psychiatric Diseases[Title/Abstract])) OR (Psychiatric Disease[Title/Abstract])) OR (Mental Illness[Title/Abstract])) OR (Illness, Mental[Title/Abstract])) OR (Mental Illnesses[Title/Abstract])) OR (Psychiatric Disorders[Title/Abstract])) OR (Psychiatric Disorder[Title/Abstract])) OR (Behavior Disorders[Title/Abstract])) OR (Diagnosis, Psychiatric[Title/Abstract])) OR (Psychiatric Diagnosis[Title/Abstract])) OR (Mental Disorders, Severe[Title/Abstract])) OR (Mental Disorder, Severe[Title/Abstract])) OR (Severe Mental Disorder[Title/Abstract])) OR (Severe Mental Disorders[Title/Abstract]) | 1,460,250 |
| #5 | ((((((((((((((((((((((((((Cognitive Disorders[MeSH Terms])) OR (Cognitive Dysfunctions[Title/Abstract])) OR (Dysfunction, Cognitive[Title/Abstract])) OR (Dysfunctions, Cognitive[Title/Abstract])) OR (Cognitive Impairments[Title/Abstract])) OR (Cognitive Impairment[Title/Abstract])) OR (Impairment, Cognitive[Title/Abstract])) OR (Impairments, Cognitive[Title/Abstract])) OR (Cognitive Disorder[Title/Abstract])) OR (Cognitive Disorders[Title/Abstract])) OR (Disorder, Cognitive[Title/Abstract])) OR (Disorders, Cognitive[Title/Abstract])) OR (Mild Cognitive Impairment[Title/Abstract])) OR (Cognitive Impairment, Mild[Title/Abstract])) OR (Cognitive Impairments, Mild[Title/Abstract])) OR (Impairment, Mild Cognitive[Title/Abstract])) OR (Impairments, Mild Cognitive[ Title/Abstract])) OR (Mild Cognitive Impairments[Title/Abstract])) OR (Cognitive Decline[Title/Abstract])) OR (Cognitive Declines[Title/Abstract])) OR (Decline, Cognitive[Title/Abstract])) OR (Declines, Cognitive[Title/Abstract])) OR (Mental Deterioration[Title/Abstract])) OR (Deterioration, Mental[Title/Abstract])) OR (Deteriorations, Mental[Title/Abstract])) OR (Mental Deteriorations[Title/Abstract]) | 130,603 |
| #4 | ((((((((((((Dementia[MeSH Terms]) OR (Dementias[Title/Abstract])) OR (Amentia[Title/Abstract])) OR (Amentias[Title/Abstract])) OR (Senile Paranoid Dementia[Title/Abstract])) OR (Dementias, Senile Paranoid[Title/Abstract])) OR (Paranoid Dementia, Senile[Title/Abstract])) OR (Paranoid Dementias, Senile[Title/Abstract])) OR (Senile Paranoid Dementias[Title/Abstract])) OR (Familial Dementia[Title/Abstract])) OR (Dementia, Familial[Title/Abstract])) OR (Dementias, Familial[Title/Abstract])) OR (Familial Dementias[Title/Abstract]) | 203,537 |
| #3 | (((((((((((((((((((((((Neurocognitive Disorders[MeSH Terms])) OR (Disorder, Neurocognitive[Title/Abstract])) OR (Disorder, Neurocognitive[Title/Abstract])) OR (Disorder, Neurocognitive[Title/Abstract])) OR (Disorder, Neurocognitive[Title/Abstract])) OR (Clerambault Syndrome[Title/Abstract])) OR (Organic Mental Disorders, Psychotic[Title/Abstract])) OR (Delirium, Dementia, Amnestic, Cognitive Disorders[Title/Abstract])) OR (Mental Disorders, Organic[Title/Abstract])) OR (Disorders, Organic Mental[Title/Abstract])) OR (Mental Disorder, Organic[Title/Abstract])) OR (Organic Mental Disorder[Title/Abstract])) OR (Organic Mental Disorders[Title/Abstract])) OR (Mild Neurocognitive Disorder[Title/Abstract])) OR (Disorder, Mild Neurocognitive[Title/Abstract])) OR (Disorders, Mild Neurocognitive[Title/Abstract])) OR (Mild Neurocognitive Disorders[Title/Abstract])) OR (Neurocognitive Disorder, Mild[Title/Abstract])) OR (Neurocognitive Disorders, Mild[Title/Abstract])) OR (Organic Brain Syndrome, Nonpsychotic[Title/Abstract])) OR (Nonpsychotic Organic Brain Syndrome[Title/Abstract])) OR (Psychoses, Traumatic[Title/Abstract])) OR (Traumatic Psychoses[Title/Abstract]) | 306,769 |
| #2 | ((((((Cognition[MeSH Terms])) OR (Cognitions[Title/Abstract])) OR (Cognitive Function[Title/Abstract])) OR (Cognitive Functions[Title/Abstract])) OR (Function, Cognitive[Title/Abstract])) OR (Functions, Cognitive[Title/Abstract]) | 245,837 |
| #1 | ((((((((Delirium[MeSH Terms])) OR (Subacute Delirium[Title/Abstract])) OR (Delirium, Subacute[Title/Abstract])) OR (Deliriums, Subacute[Title/Abstract])) OR (Subacute Deliriums[Title/Abstract])) OR (Delirium of Mixed Origin[Title/Abstract])) OR (Mixed Origin Delirium[Title/Abstract])) OR (Mixed Origin Deliriums[Title/Abstract]) | 12,412 |

1. **Embase**

| **No.** | **Query** | **Results** |
| --- | --- | --- |
| #17 | #15 AND #16 | 1,163 |
| #16 | #13 OR #14 | 17,511 |
| #15 | #1 OR #2 OR #3 OR #4 OR #5 OR #6 OR #7 OR #8 OR #9 OR #10 OR #11 OR #12 | 5,142,559 |
| #14 | 'xenon':ab,kw,ti | 9,555 |
| #13 | 'xenon'/exp OR xenon | 17,511 |
| #12 | 'disorder, neurocognitive':ab,kw,ti OR 'clerambault syndrome':ab,kw,ti OR 'organic mental disorders, psychotic':ab,kw,ti OR 'delirium, dementia, amnestic, cognitive disorders':ab,kw,ti OR 'mental disorders, organic':ab,kw,ti OR 'disorders, organic mental':ab,kw,ti OR 'mental disorder, organic':ab,kw,ti OR 'organic mental disorder':ab,kw,ti OR 'organic mental disorders':ab,kw,ti OR 'mild neurocognitive disorder':ab,kw,ti OR 'disorder, mild neurocognitive':ab,kw,ti OR 'disorders, mild neurocognitive':ab,kw,ti OR 'mild neurocognitive disorders':ab,kw,ti OR 'neurocognitive disorder, mild':ab,kw,ti OR 'neurocognitive disorders, mild':ab,kw,ti OR 'organic brain syndrome, nonpsychotic':ab,kw,ti OR 'nonpsychotic organic brain syndrome':ab,kw,ti OR 'psychoses, traumatic':ab,kw,ti OR 'traumatic psychoses':ab,kw,ti | 1,285 |
| #11 | 'neurocognitive disorders'/exp OR 'neurocognitive disorders' | 981,545 |
| #10 | 'dementias':ab,kw,ti OR 'amentia':ab,kw,ti OR 'amentias':ab,kw,ti OR 'senile paranoid dementia':ab,kw,ti OR 'dementias, senile paranoid':ab,kw,ti OR 'paranoid dementia, senile':ab,kw,ti OR 'paranoid dementias, senile':ab,kw,ti OR 'senile paranoid dementias':ab,kw,ti OR 'familial dementia':ab,kw,ti OR 'dementia, familial':ab,kw,ti OR 'dementias, familial':ab,kw,ti OR 'familial dementias':ab,kw,ti | 9,893 |
| #9 | 'dementia'/exp OR 'dementia' | 482,113 |
| #8 | 'cognitive dysfunctions':ab,kw,ti OR 'dysfunction, cognitive':ab,kw,ti OR 'dysfunctions, cognitive':ab,kw,ti OR 'cognitive impairments':ab,kw,ti OR 'cognitive impairment':ab,kw,ti OR 'impairment, cognitive':ab,kw,ti OR 'impairments, cognitive':ab,kw,ti OR 'cognitive disorder':ab,kw,ti OR 'cognitive disorders':ab,kw,ti OR 'disorder, cognitive':ab,kw,ti OR 'disorders, cognitive':ab,kw,ti OR 'mild cognitive impairment':ab,kw,ti OR 'cognitive impairment, mild':ab,kw,ti OR 'cognitive impairments, mild':ab,kw,ti OR 'impairment, mild cognitive':ab,kw,ti OR 'impairments, mild cognitive':ab,kw,ti OR 'mild cognitive impairments':ab,kw,ti OR 'cognitive decline':ab,kw,ti OR 'cognitive declines':ab,kw,ti OR 'decline, cognitive':ab,kw,ti OR 'declines, cognitive':ab,kw,ti OR 'mental deterioration':ab,kw,ti OR 'deterioration, mental':ab,kw,ti OR 'deteriorations, mental':ab,kw,ti OR 'mental deteriorations':ab,kw,ti | 178,703 |
| #7 | 'cognitive disorders'/exp OR 'cognitive disorders' | 602,668 |
| #6 | 'mental disorder':ab,kw,ti OR 'psychiatric illness':ab,kw,ti OR 'psychiatric  illnesses':ab,kw,ti OR 'psychiatric diseases':ab,kw,ti OR 'psychiatric disease':ab,kw,ti OR 'mental illness':ab,kw,ti OR 'illness, mental':ab,kw,ti OR 'mental illnesses':ab,kw,ti OR 'psychiatric disorders':ab,kw,ti OR 'psychiatric disorder':ab,kw,ti OR 'behavior disorders':ab,kw,ti OR 'diagnosis, psychiatric':ab,kw,ti OR 'psychiatric diagnosis':ab,kw,ti OR 'mental disorders, severe':ab,kw,ti OR 'mental disorder, severe':ab,kw,ti OR 'severe mental disorder':ab,kw,ti OR 'severe mental disorders':ab,kw,ti | 167,460 |
| #5 | 'mental disorders'/exp OR 'mental disorders' | 2,754,204 |
| #4 | 'cognitions':ab,kw,ti OR 'cognitive function':ab,kw,ti OR 'cognitive functions':ab,kw,ti OR 'function, cognitive':ab,kw,ti OR 'functions, cognitive':ab,kw,ti | 106,777 |
| #3 | 'cognition'/exp OR cognition | 3,022,811 |
| #2 | 'subacute delirium':ab,kw,ti OR 'delirium, subacute':ab,kw,ti OR 'deliriums, subacute':ab,kw,ti OR 'subacute deliriums':ab,kw,ti OR 'delirium of mixed origin':ab,kw,ti OR 'mixed origin delirium':ab,kw,ti OR 'mixed origin deliriums':ab,kw,ti | 19 |
| #1 | 'delirium'/exp OR delirium | 46,748 |

1. **Cochrane**

| **No.** | **Query** | **Results** |
| --- | --- | --- |
| #9 | #7 AND #8 | 43 |
| #8 | xenon:ab,kw,ti OR xenon:ab,kw,ti | 554 |
| #7 | #1 OR #2 OR #3 OR #4 OR #5 OR #6 | 81,266 |
| #6 | “Neurocognitive Disorders”:ab,kw,ti OR “Disorder, Neurocognitive”:ab,kw,ti OR “Disorder, Neurocognitive”:ab,kw,ti OR “Disorder, Neurocognitive”:ab,kw,ti OR “Disorder, Neurocognitive”:ab,kw,ti OR “Clerambault Syndrome”:ab,kw,ti OR “Organic Mental Disorders, Psychotic”:ab,kw,ti OR “Delirium, Dementia, Amnestic, Cognitive Disorders”:ab,kw,ti OR “Mental Disorders, Organic”:ab,kw,ti OR “Disorders, Organic Mental”:ab,kw,ti OR “Mental Disorder, Organic”:ab,kw,ti OR “Organic Mental Disorder”:ab,kw,ti OR “Organic Mental Disorders”:ab,kw,ti OR “Mild Neurocognitive Disorder”:ab,kw,ti OR “Disorder, Mild Neurocognitive”:ab,kw,ti OR “Disorders, Mild Neurocognitive”:ab,kw,ti OR “Mild Neurocognitive Disorders”:ab,kw,ti OR “Neurocognitive Disorder, Mild”:ab,kw,ti OR “Neurocognitive Disorders, Mild”:ab,kw,ti OR “Organic Brain Syndrome, Nonpsychotic”:ab,kw,ti OR “Nonpsychotic Organic Brain Syndrome”:ab,kw,ti OR “Psychoses, Traumatic”:ab,kw,ti OR “Traumatic Psychoses”:ab,kw,ti | 502 |
| #5 | Dementia:ab,kw,ti OR Dementias:ab,kw,ti OR Amentia:ab,kw,ti OR Amentias:ab,kw,ti OR “Senile Paranoid Dementia”:ab,kw,ti OR “Dementias, Senile Paranoid”:ab,kw,ti OR “Paranoid Dementia, Senile”:ab,kw,ti OR “Paranoid Dementias, Senile”:ab,kw,ti OR “Senile Paranoid Dementias”:ab,kw,ti OR “Familial Dementia”:ab,kw,ti OR “Dementia, Familial”:ab,kw,ti OR “Dementias, Familial”:ab,kw,ti OR “Familial Dementias”:ab,kw,ti | 16,110 |
| #4 | “Cognitive Disorders”:ab,kw,ti OR “Cognitive Dysfunctions”:ab,kw,ti OR “Dysfunction, Cognitive”:ab,kw,ti OR “Dysfunctions, Cognitive”:ab,kw,ti OR “Cognitive Impairments”:ab,kw,ti OR “Cognitive Impairment”:ab,kw,ti OR “Impairment, Cognitive”:ab,kw,ti OR “Impairments, Cognitive”:ab,kw,ti OR “Cognitive Disorder”:ab,kw,ti OR “Cognitive Disorders”:ab,kw,ti OR “Disorder, Cognitive”:ab,kw,ti OR “Disorders, Cognitive”:ab,kw,ti OR “Mild Cognitive Impairment”:ab,kw,ti OR “Cognitive Impairment, Mild”:ab,kw,ti OR “Cognitive Impairments, Mild”:ab,kw,ti OR “Impairment, Mild Cognitive”:ab,kw,ti OR “Impairments, Mild Cognitive”:ab,kw,ti OR “Mild Cognitive Impairments”:ab,kw,ti OR “Cognitive Decline”:ab,kw,ti OR “Cognitive Declines”:ab,kw,ti OR “Decline, Cognitive”:ab,kw,ti OR “Declines, Cognitive”:ab,kw,ti OR “Mental Deterioration”:ab,kw,ti OR “Deterioration, Mental”:ab,kw,ti OR “Deteriorations, Mental”:ab,kw,ti OR “Mental Deteriorations”:ab,kw,ti | 15,622 |
| #3 | “Mental Disorders”:ab,kw,ti OR “Mental Disorder”:ab,kw,ti OR “Psychiatric Illness”:ab,kw,ti OR “Psychiatric Illnesses”:ab,kw,ti OR “Psychiatric Diseases”:ab,kw,ti OR “Psychiatric Disease”:ab,kw,ti OR “Mental Illness”:ab,kw,ti OR “Illness, Mental”:ab,kw,ti OR “Mental Illnesses”:ab,kw,ti OR “Psychiatric Disorders”:ab,kw,ti OR “Psychiatric Disorder”:ab,kw,ti OR “Behavior Disorders”:ab,kw,ti OR “Diagnosis, Psychiatric”:ab,kw,ti OR “Psychiatric Diagnosis”:ab,kw,ti OR “Mental Disorders, Severe”:ab,kw,ti OR “Mental Disorder, Severe”:ab,kw,ti OR “Severe Mental Disorder”:ab,kw,ti OR “Severe Mental Disorders”:ab,kw,ti | 22,394 |
| #2 | Cognition:ab,kw,ti OR Cognitions:ab,kw,ti OR “Cognitive Function”:ab,kw,ti OR “Cognitive Functions”:ab,kw,ti OR “Function, Cognitive”:ab,kw,ti OR “Functions, Cognitive”:ab,kw,ti | 41,063 |
| #1 | Delirium:ab,kw,ti OR “Subacute Delirium”:ab,kw,ti OR “Delirium, Subacute”:ab,kw,ti OR “Deliriums, Subacute”:ab,kw,ti OR “Subacute Deliriums”:ab,kw,ti OR “Delirium of Mixed Origin”:ab,kw,ti OR “Mixed Origin Delirium”:ab,kw,ti OR “Mixed Origin Deliriums”:ab,kw,ti | 5,011 |

1. **Web of Science**

| **No.** | **Query** | **Results** |
| --- | --- | --- |
| #1 | TS=(Delirium) OR TI=(Subacute Delirium OR Delirium, Subacute OR Deliriums, Subacute OR Subacute Deliriums OR Delirium of Mixed Origin OR Mixed Origin Delirium OR Mixed Origin Deliriums) OR AB=(Subacute Delirium OR Delirium, Subacute OR Deliriums, Subacute OR Subacute Deliriums OR Delirium of Mixed Origin OR Mixed Origin Delirium OR Mixed Origin Deliriums) OR KP=(Subacute Delirium OR Delirium, Subacute OR Deliriums, Subacute OR Subacute Deliriums OR Delirium of Mixed Origin OR Mixed Origin Delirium OR Mixed Origin Deliriums) | 23,956 |
| #2 | TS=(Cognition) OR TI=(Cognitions OR Cognitive Function OR Cognitive Functions OR Function, Cognitive OR Functions, Cognitive) | 212,340 |
| #3 | TS=(Mental Disorders) OR TI=(Mental Disorder OR Psychiatric Illness OR Psychiatric Illnesses OR Psychiatric Diseases OR Psychiatric Disease OR Mental Illness OR Illness, Mental OR Mental Illnesses OR Psychiatric Disorders OR Psychiatric Disorder OR Behavior Disorders OR Diagnosis, Psychiatric OR Psychiatric Diagnosis OR Mental Disorders, Severe OR Mental Disorder, Severe OR Severe Mental Disorder OR Severe Mental Disorders) OR AB=(Mental Disorder OR Psychiatric Illness OR Psychiatric Illnesses OR Psychiatric Diseases OR Psychiatric Disease OR Mental Illness OR Illness, Mental OR Mental Illnesses OR Psychiatric Disorders OR Psychiatric Disorder OR Behavior Disorders OR Diagnosis, Psychiatric OR Psychiatric Diagnosis OR Mental Disorders, Severe OR Mental Disorder, Severe OR Severe Mental Disorder OR Severe Mental Disorders) OR KP=(Mental Disorder OR Psychiatric Illness OR Psychiatric Illnesses OR Psychiatric Diseases OR Psychiatric Disease OR Mental Illness OR Illness, Mental OR Mental Illnesses OR Psychiatric Disorders OR Psychiatric Disorder OR Behavior Disorders OR Diagnosis, Psychiatric OR Psychiatric Diagnosis OR Mental Disorders, Severe OR Mental Disorder, Severe OR Severe Mental Disorder OR Severe Mental Disorders) | 451,804 |
| #4 | TS=(Cognitive Disorders) OR TI=(Cognitive Dysfunctions OR Dysfunction, Cognitive OR Dysfunctions, Cognitive OR Cognitive Impairments OR Cognitive Impairment OR Impairment, Cognitive OR Impairments, Cognitive OR Cognitive Disorder OR Cognitive Disorders OR Disorder, Cognitive OR Disorders, Cognitive OR Mild Cognitive Impairment OR Cognitive Impairment, Mild OR Cognitive Impairments, Mild OR Impairment, Mild Cognitive OR Impairments, Mild Cognitive OR Mild Cognitive Impairments OR Cognitive Decline OR Cognitive Declines OR Decline, Cognitive OR Declines, Cognitive OR Mental Deterioration OR Deterioration, Mental OR Deteriorations, Mental OR Mental Deteriorations) OR AB=(Cognitive Dysfunctions OR Dysfunction, Cognitive OR Dysfunctions, Cognitive OR Cognitive Impairments OR Cognitive Impairment OR Impairment, Cognitive OR Impairments, Cognitive OR Cognitive Disorder OR Cognitive Disorders OR Disorder, Cognitive OR Disorders, Cognitive OR Mild Cognitive Impairment OR Cognitive Impairment, Mild OR Cognitive Impairments, Mild OR Impairment, Mild Cognitive OR Impairments, Mild Cognitive OR Mild Cognitive Impairments OR Cognitive Decline OR Cognitive Declines OR Decline, Cognitive OR Declines, Cognitive OR Mental Deterioration OR Deterioration, Mental OR Deteriorations, Mental OR Mental Deteriorations) OR KP=(Cognitive Dysfunctions OR Dysfunction, Cognitive OR Dysfunctions, Cognitive OR Cognitive Impairments OR Cognitive Impairment OR Impairment, Cognitive OR Impairments, Cognitive OR Cognitive Disorder OR Cognitive Disorders OR Disorder, Cognitive OR Disorders, Cognitive OR Mild Cognitive Impairment OR Cognitive Impairment, Mild OR Cognitive Impairments, Mild OR Impairment, Mild Cognitive OR Impairments, Mild Cognitive OR Mild Cognitive Impairments OR Cognitive Decline OR Cognitive Declines OR Decline, Cognitive OR Declines, Cognitive OR Mental Deterioration OR Deterioration, Mental OR Deteriorations, Mental OR Mental Deteriorations) | 312,347 |
| #5 | TS=(Dementia) OR TI=(Dementias OR Amentia OR Amentias OR Senile Paranoid Dementia OR Dementias, Senile Paranoid OR Paranoid Dementia, Senile OR Paranoid Dementias, Senile OR Senile Paranoid Dementias OR Familial Dementia OR Dementia, Familial OR Dementias, Familial OR Familial Dementias) OR AB=(Dementias OR Amentia OR Amentias OR Senile Paranoid Dementia OR Dementias, Senile Paranoid OR Paranoid Dementia, Senile OR Paranoid Dementias, Senile OR Senile Paranoid Dementias OR Familial Dementia OR Dementia, Familial OR Dementias, Familial OR Familial Dementias) OR KP=(Dementias OR Amentia OR Amentias OR Senile Paranoid Dementia OR Dementias, Senile Paranoid OR Paranoid Dementia, Senile OR Paranoid Dementias, Senile OR Senile Paranoid Dementias OR Familial Dementia OR Dementia, Familial OR Dementias, Familial OR Familial Dementias) | 200,488 |
| #6 | TS=(Neurocognitive Disorders) OR TI=(Disorder, Neurocognitive OR Disorder, Neurocognitive OR Disorder, Neurocognitive OR Disorder, Neurocognitive OR Clerambault Syndrome OR Organic Mental Disorders, Psychotic OR Delirium, Dementia, Amnestic, Cognitive Disorders OR Mental Disorders, Organic OR Disorders, Organic Mental OR Mental Disorder, Organic OR Organic Mental Disorder OR Organic Mental Disorders OR Mild Neurocognitive Disorder OR Disorder, Mild Neurocognitive OR Disorders, Mild Neurocognitive OR Mild Neurocognitive Disorders OR Neurocognitive Disorder, Mild OR Neurocognitive Disorders, Mild OR Organic Brain Syndrome, Nonpsychotic OR Nonpsychotic Organic Brain Syndrome OR Psychoses, Traumatic OR Traumatic Psychoses) OR AB=(Disorder, Neurocognitive OR Disorder, Neurocognitive OR Disorder, Neurocognitive OR Disorder, Neurocognitive OR Clerambault Syndrome OR Organic Mental Disorders, Psychotic OR Delirium, Dementia, Amnestic, Cognitive Disorders OR Mental Disorders, Organic OR Disorders, Organic Mental OR Mental Disorder, Organic OR Organic Mental Disorder OR Organic Mental Disorders OR Mild Neurocognitive Disorder OR Disorder, Mild Neurocognitive OR Disorders, Mild Neurocognitive OR Mild Neurocognitive Disorders OR Neurocognitive Disorder, Mild OR Neurocognitive Disorders, Mild OR Organic Brain Syndrome, Nonpsychotic OR Nonpsychotic Organic Brain Syndrome OR Psychoses, Traumatic OR Traumatic Psychoses) OR KP=(Disorder, Neurocognitive OR Disorder, Neurocognitive OR Disorder, Neurocognitive OR Disorder, Neurocognitive OR Clerambault Syndrome OR Organic Mental Disorders, Psychotic OR Delirium, Dementia, Amnestic, Cognitive Disorders OR Mental Disorders, Organic OR Disorders, Organic Mental OR Mental Disorder, Organic OR Organic Mental Disorder OR Organic Mental Disorders OR Mild Neurocognitive Disorder OR Disorder, Mild Neurocognitive OR Disorders, Mild Neurocognitive OR Mild Neurocognitive Disorders OR Neurocognitive Disorder, Mild OR Neurocognitive Disorders, Mild OR Organic Brain Syndrome, Nonpsychotic OR Nonpsychotic Organic Brain Syndrome OR Psychoses, Traumatic OR Traumatic Psychoses) | 16,553 |
| #7 | #6 OR #5 OR #4 OR #3 OR #2 OR #1 | 985,389 |
| #8 | TS=(xenon) OR TI=(xenon) OR AB=(xenon) OR KP=(xenon) | 23,256 |
| #9 | #8 AND #7 | 208 |
