# Supplementary material for: Effects of xenon anesthesia on postoperative neurocognitive disorders: a systematic review and meta-analysis
Source: BMC Anesthesiol. 2023 Nov 9;23:366. doi: 10.1186/s12871-023-02316-5 (PMC10634138; doi:10.1186/s12871-023-02316-5)
Supplement: Supplementary file 2 — Additional file 2. Author's definition of each outcome and the anaesthesia induction program. [file 12871_2023_2316_MOESM2_ESM.docx]

**Additional file 2**

**1.Author's definition of each outcome**

(1) Rasmussen 2006

1) Definition of Postoperative neurocognitive dysfunction (PND): Postoperative neurocognitive dysfunction (PND): Patients with Z-score above 2 in 2 tests, Composite Z-score above 2, and 7 parameters and logarithmic scale were considered as the presence of PND according to ISPOCD neuropsychological test battery.

2) The timing of neurocognitive evaluations: 1–5 days before surgery, at discharge 3–5 days after surgery, and 10–14 weeks after surgery.

(2) Coburn 2007

1) Definition of PND: Postoperative neurocognitive dysfunction (PND): The computerized Test for Attentional Performance (TAP, Version 1.7; Psytest, 2002)

2) The timing of neurocognitive evaluations: 12–24 h before operation, 6–12 and 66–72 h after operation.

(3) Stuttmann 2010

1) Definition of PND: A syndrome short test with a sum score of 3-4 points indicates suspicion of cognitive dysfunction; 5-8 points suggests light cognitive dysfunction and 9-13 points suggests the beginning of a psycho-organic syndrome.

2) The timing of neurocognitive evaluations: on the day before operation; post-operatively at 60- and 180-minutes post extubation.

(4) Bronco 2010

1) Definition of PND: A 2.5-point difference on Short Orientation Memory Concentration Test (SOMCT) scores at 30 min was considered to be clinically significant.

2) The timing of neurocognitive evaluations: 30 min before induction, 30 min after extubation and 1 h after extubation.

(5) Cremer 2011

1) Definition of PND: A patient with a 20% decrease in at least 20% of all tests (including the subtest “Alertness” of the computerized Test for Attentional Performance (TAP, Version 1.7; Psytest, 2002) and additional Paper-Pencil-Tests) was considered to be suffering from PND.

2) The timing of neurocognitive evaluations: 12-24 h before the operation, 6-12 and 66-72 h after surgery.

(6) Stoppe 2013

1) Definition of PND: CAM-ICU score

2) Definition of Adverse event (AE): AE was defined as any undesirable and unintended sign or symptom occurring in a patient during the observation period, not necessarily in any causal relationship with the study treatment.

3) The timing of neurocognitive evaluations: 1 day before surgery (baseline); After operation, CAM-ICU score was assessed daily.

(7) Al tmimi 2015

1) Definition of PND: The Confusion Assessment Method (CAM) for patients in ward, or the confusion assessment method adapted for patients in the ICU (CAM-ICU: delirium is diagnosed when Features 1 and 2 are positive, along with either Feature 3 or Feature 4)^1^.

2) Definition of Adverse event (AE): Defined as ‘any untoward medical occurrence in a patient or clinical investigation subject administered a pharmaceutical product and which does not necessarily have to have a causal relationship with this treatment.’

3) Definition of Myocardial infarction: Defined as the occurrence of a new Q wave in addition to an increase in troponin T exceeding the 99th percentile of the upper reference level in the early postoperative period or any episode of chest pain with a typical increase of cardiac enzyme

4) The timing of neurocognitive evaluations: During the ICU stay, patients were assessed daily for the presence of POD by trained research nurses (blinded to the group assignment) using the CAM-ICU; After transferal to the ward, patients were also screened daily for the presence of POD, until postoperative day 9, using the CAM.

(8) Coburn 2018

1) Definition of PND: According to a previous reference (when the presence of both the first and the second criteria and of either the third or the fourth criterion in the CAM algorithm, which was considered as the presence of delirium).

2) The timing of neurocognitive evaluations: postoperative day five through discharge

(9) Al tmimi 2020

1) Definition of PND: A subject was defined as POD-positive if at least one POD episode occurred within the first 5 postoperative days, as indicated by a positive 3-Minute Diagnostic Interview for Confusion Assessment Method (CAM) (3D-CAM) or CAM-ICU assessment.

2) The timing of neurocognitive evaluations: Daily POD screening was performed by trained research nurses who were blinded to group allocation.

**2.The anaesthesia induction program**

| **Authors** | **Induction drugs** | **Dose** |
| --- | --- | --- |
| Rasmussen 2006 | Propofol | 1 - 2 mg/kg |
| Coburn 2007 | Propofol + remifentanil | 2 mg/kg + 0.5 μg/kg over 60 s |
| Bronco 2010 | Propofol | 2 - 3 mg/kg |
| Stuttmann 2010 | Propofol + Fentanyl + Rocuronium Bromide | 1-2 mg/kg + 0.003 mg/kg + 0.6 mg/kg |
| Cremer 2011 | Propofol + remifentanil | 2 mg/kg + 0.5 μg/kg over 60 s |
| Stoppe 2013 | Propofol + Sufentanil | 1 mg/kg + 0.5–1 µg/kg |
| Al tmimi 2015 | Propofol + Sufentanil | 0.5–1 mg/kg + 0.25–0.5 µg/kg |
| Coburn 2018 | Propofol | 1 - 2 mg/kg |
| Al tmimi 2020 | Remifentanil | 0.5 mg/kg/min |
