# Supplementary figures and images for: Effects of xenon anesthesia on postoperative neurocognitive disorders: a systematic review and meta-analysis
Source: BMC Anesthesiol. 2023 Nov 9;23:366. doi: 10.1186/s12871-023-02316-5 (PMC10634138; doi:10.1186/s12871-023-02316-5)

**Additional file 3**

**Flow diagram of the literature search**


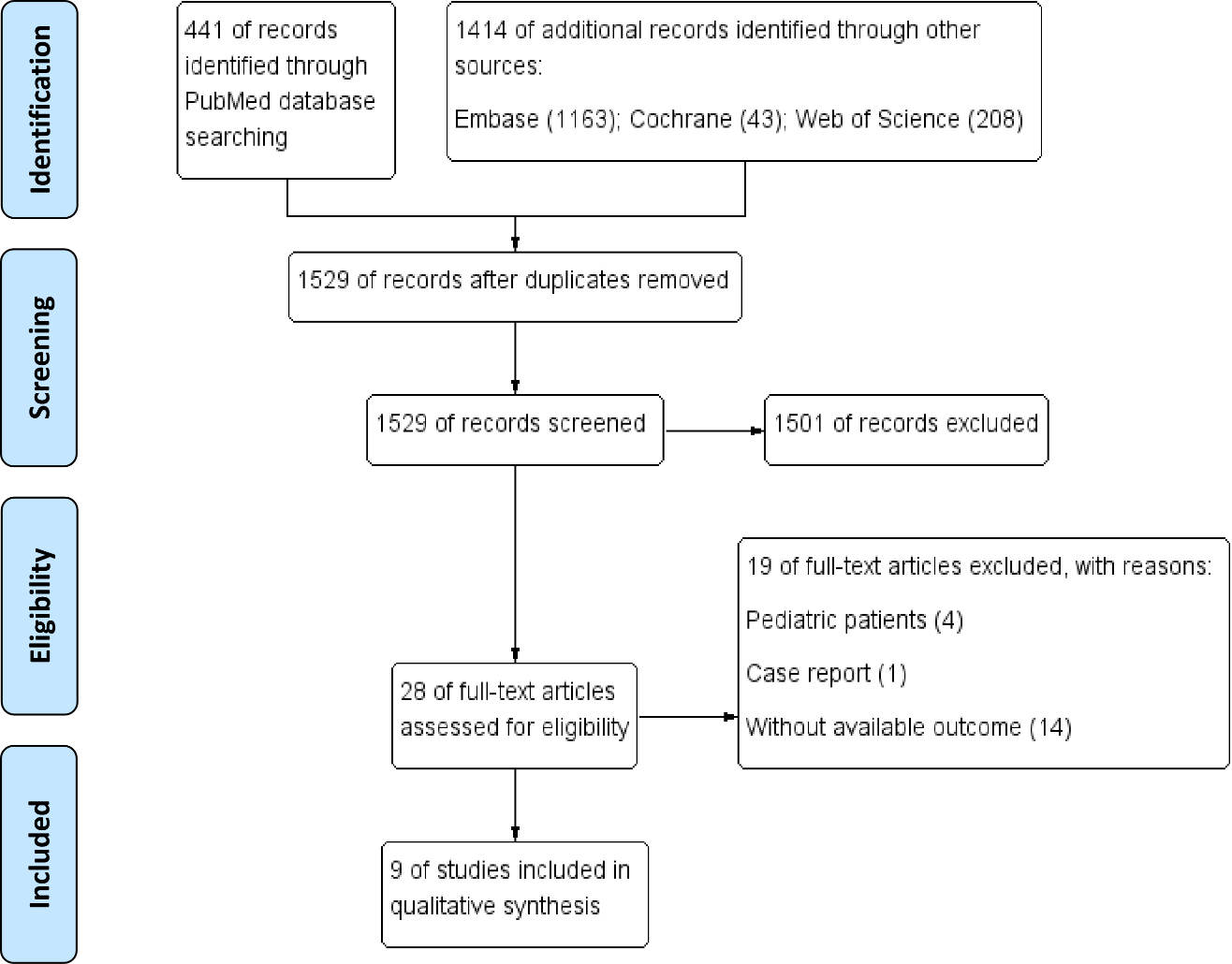

Supplement: Supplementary file 3 — Additional file 3. Flow diagram of the literature search. [file 12871_2023_2316_MOESM3_ESM.docx]

**Additional file 7:** Forest plot of the pooled analysis of postoperative complications


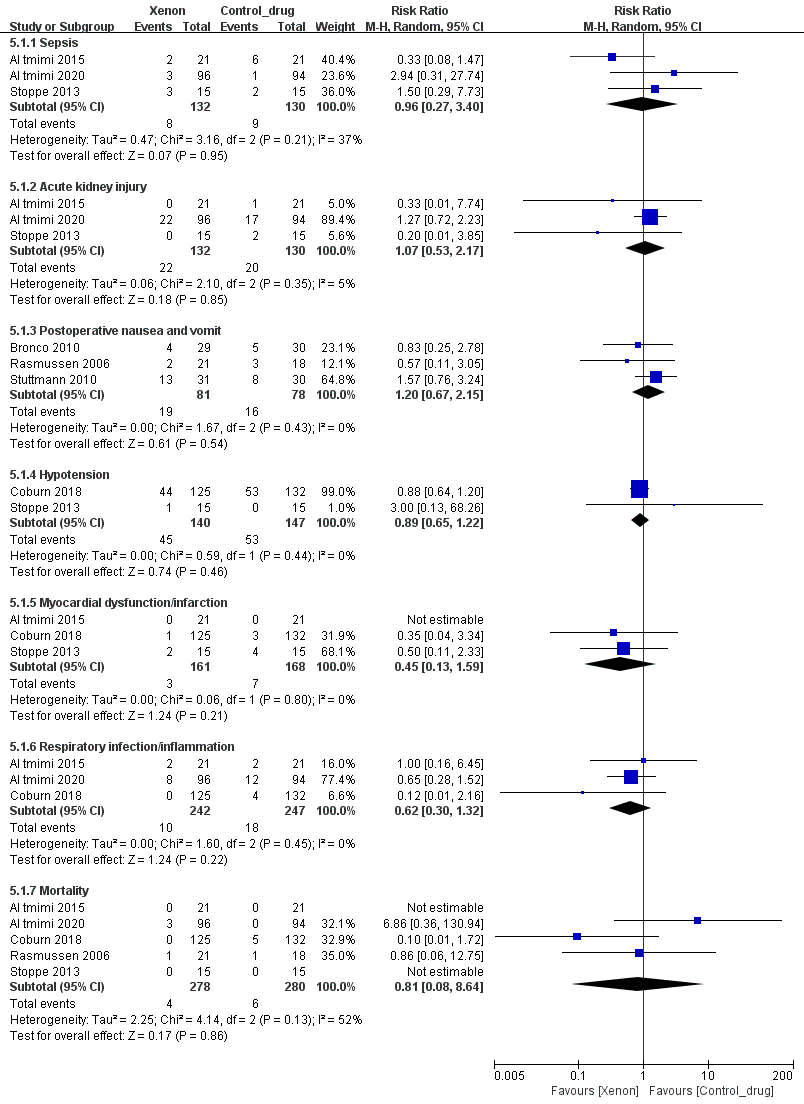

Supplement: Supplementary file 7 — Additional file 7. Forest plot of the pooled analysis of postoperative complications. [file 12871_2023_2316_MOESM7_ESM.docx]
