# Supplementary material for: Effects of xenon anesthesia on postoperative neurocognitive disorders: a systematic review and meta-analysis
Source: BMC Anesthesiol. 2023 Nov 9;23:366. doi: 10.1186/s12871-023-02316-5 (PMC10634138; doi:10.1186/s12871-023-02316-5)
Supplement: Supplementary file 4 — Additional file 4. Risk of bias (ROB 1.0) evaluations for the included randomized-controlled trials. Risk of bias (ROB 2.0) assessment of included randomized controlled trials. [file 12871_2023_2316_MOESM4_ESM.docx]

**Additional file 4:**

**1. Risk of bias (ROB 1.0) evaluations for the included randomized-controlled trials.**
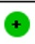
, low risk;
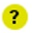
, unclear risk;
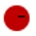
, high risk.


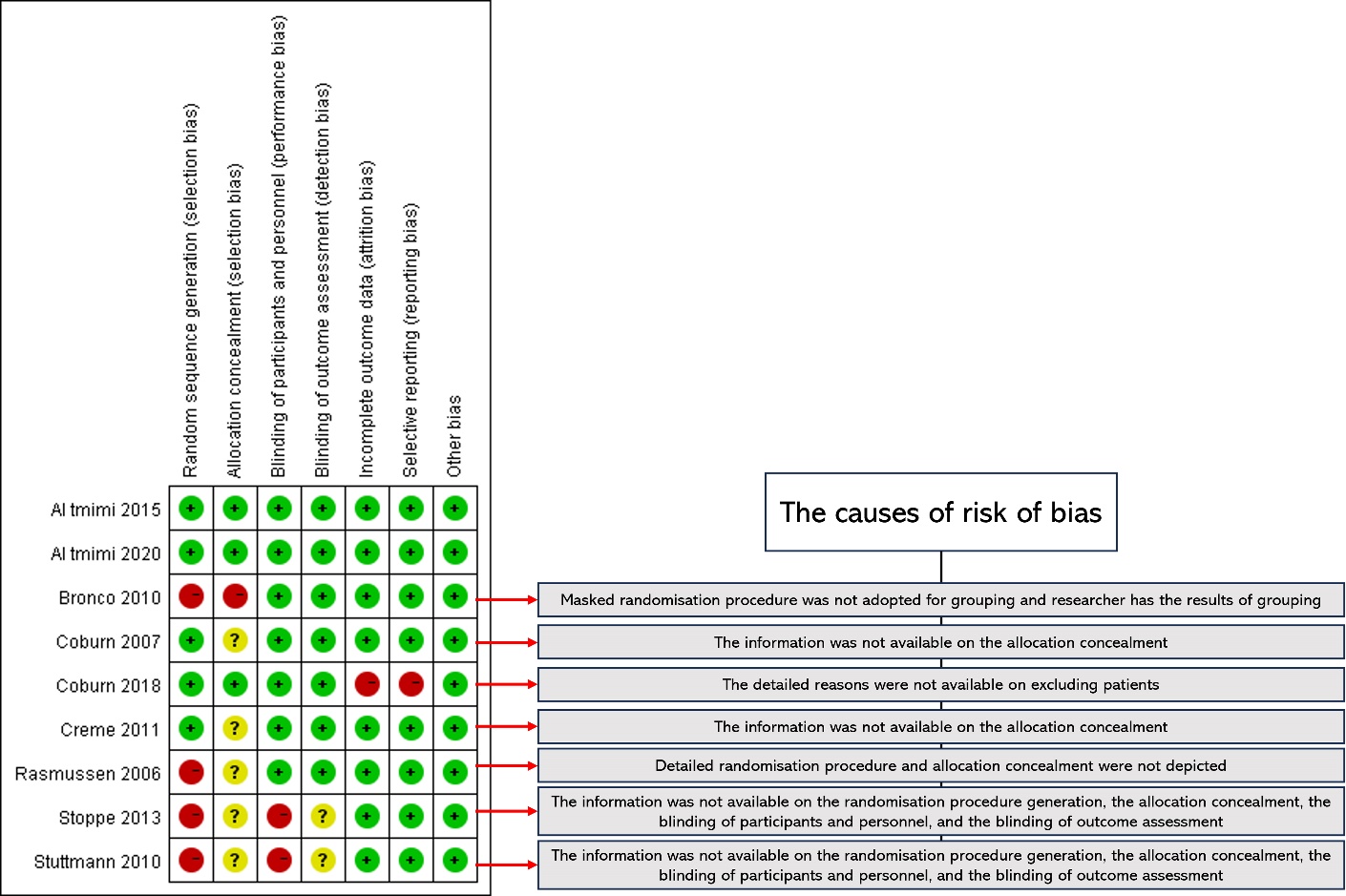


| **References** | **Domain A** | **Domain B** | **Domain C** | **Domain D** | **Domain E** | **Overall risk of bias** |
| --- | --- | --- | --- | --- | --- | --- |
| Rasmussen 2006 | Some concerns | Some concerns | Low | Low | Low | Some concerns |
| Coburn 2007 | Low | Some concerns | Low | Low | Low | Some concerns |
| Bronco 2010 | Some concerns | Some concerns | Low | Low | Low | Some concerns |
| Stuttmann 2010 | Some concerns | Some concerns | Low | Some concerns | Low | Some concerns |
| Cremer 2011 | Low | Some concerns | Low | Low | Low | Some concerns |
| Stoppe 2013 | Some concerns | Some concerns | Low | Some concerns | Low | Some concerns |
| Al tmimi 2015 | Low | Low | Low | Low | Low | Low |
| Coburn 2018 | Low | Low | Some concerns | Low | Some concerns | Some concerns |
| Al tmimi 2020 | Low | Low | Low | Low | Low | Low |
| Domains: (A) bias arising from the randomization process, (B) bias owing to deviations from intended interventions, (C) bias owing to missing outcome data, (D) bias in measurement of the outcome, (E) bias in selection of the reported result. The overall risk of bias grade was calculated by assessing the five domains [A–E]. | | | | | | |

**2. Risk of bias (ROB 2.0) assessment of included randomized controlled trials**
