# Supplementary material for: Effects of xenon anesthesia on postoperative neurocognitive disorders: a systematic review and meta-analysis
Source: BMC Anesthesiol. 2023 Nov 9;23:366. doi: 10.1186/s12871-023-02316-5 (PMC10634138; doi:10.1186/s12871-023-02316-5)

**Additional file 5:** Forest plot of the pooled analysis showing the subgroup analysis for the incidence of PND according to different surgery types (PND, postoperative cognitive dysfunction).


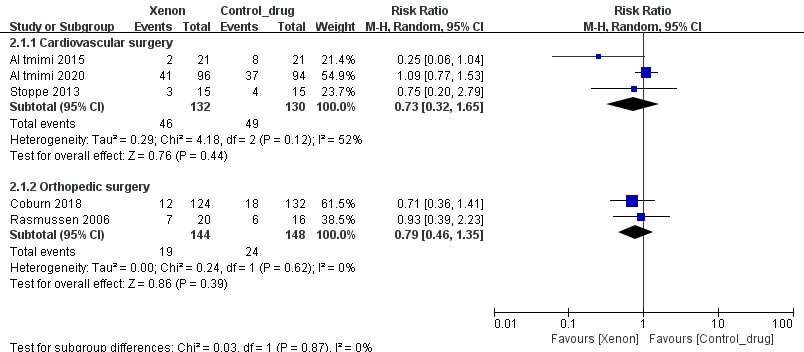

Supplement: Supplementary file 5 — Additional file 5. Forest plot of the pooled analysis showing the subgroup analysis for the incidence of PND according to different surgery types (PND, postoperative cognitive dysfunction). [file 12871_2023_2316_MOESM5_ESM.docx]
