# Supplementary material for: Effects of xenon anesthesia on postoperative neurocognitive disorders: a systematic review and meta-analysis
Source: BMC Anesthesiol. 2023 Nov 9;23:366. doi: 10.1186/s12871-023-02316-5 (PMC10634138; doi:10.1186/s12871-023-02316-5)
Supplement: Supplementary file 6 — Additional file 6. The results of sensitivity analysis. [file 12871_2023_2316_MOESM6_ESM.docx]

| Included literatures | HR (CI) | P value | I |
| --- | --- | --- | --- |
| Without omittance | 0.87 [0.61, 1.24] | 0.43 | 19% |
| Omitting Al tmimi 2015 | 0.98 [0.74, 1.30] | 0.88 | 0% |
| Omitting Al tmimi 2020 | 0.69 [0.43, 1.11] | 0.12 | 0% |
| Omitting Coburn 2018 | 0.87 [0.54, 1.41] | 0.57 | 28% |
| Omitting Rasmussen 2006 | 0.79 [0.48, 1.30] | 0.36 | 39% |
| Omitting Stoppe 2013 | 0.84 [0.54, 1.30] | 0.43 | 38% |

**Additional file 6** The results of sensitivity analysis
