# Supplementary material for: Effects of xenon anesthesia on postoperative neurocognitive disorders: a systematic review and meta-analysis
Source: BMC Anesthesiol. 2023 Nov 9;23:366. doi: 10.1186/s12871-023-02316-5 (PMC10634138; doi:10.1186/s12871-023-02316-5)

**Additional file 8:** The result of trim and fill analysis for the incidence of postoperative neurocognitive disorders


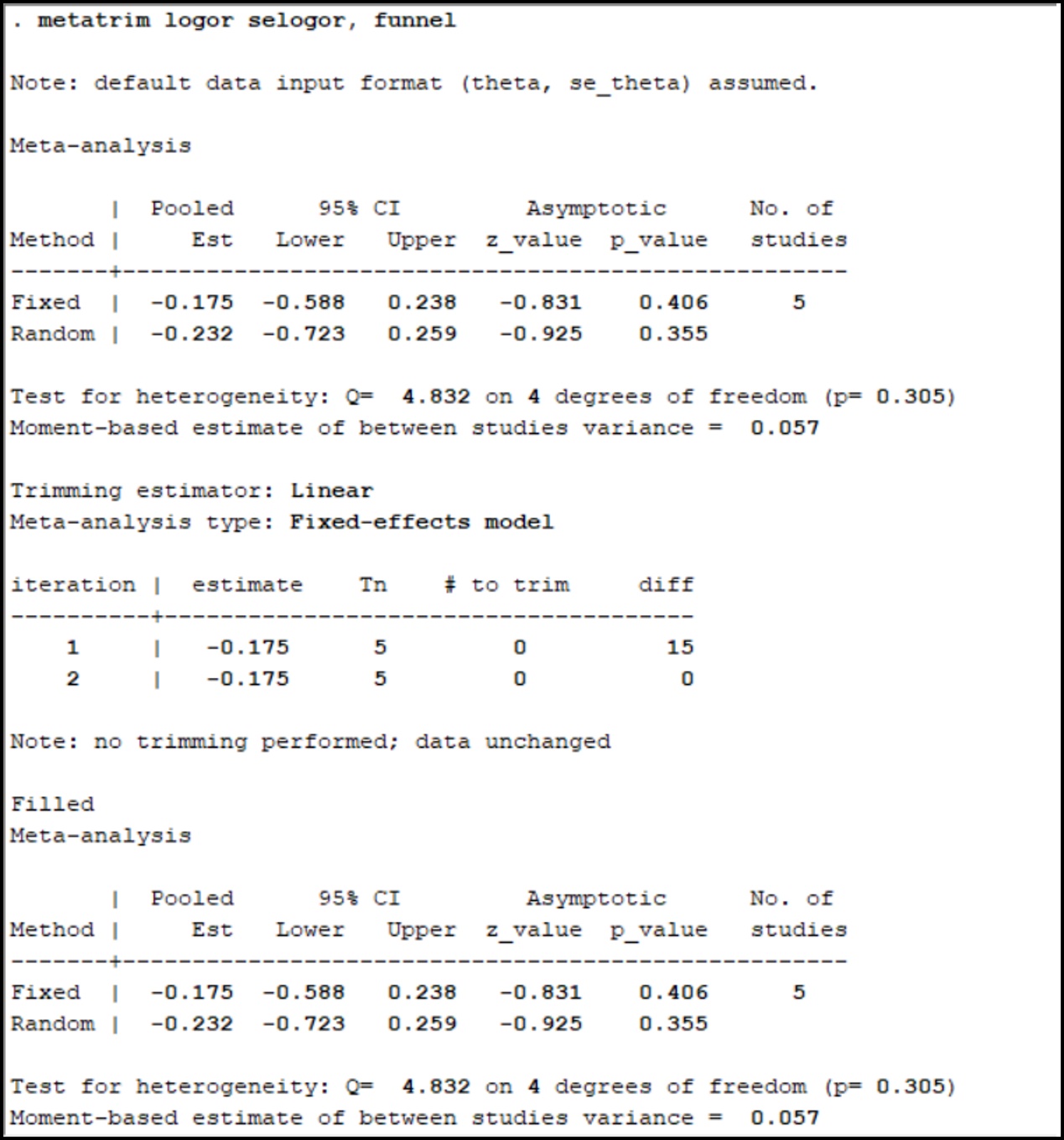

Supplement: Supplementary file 8 — Additional file 8. The result of trim and fill analysis for the incidence of postoperative neurocognitive disorders. [file 12871_2023_2316_MOESM8_ESM.docx]
